# Glycans as Biomarkers in Prostate Cancer

**DOI:** 10.3390/ijms20061389

**Published:** 2019-03-19

**Authors:** Emma Scott, Jennifer Munkley

**Affiliations:** Institute of Genetic Medicine, Newcastle University, Newcastle upon Tyne NE1 3BZ, UK; Emma.Scott@newcastle.ac.uk

**Keywords:** prostate cancer, biomarkers, liquid biopsy, glycans, glycosylation

## Abstract

Prostate cancer is the most commonly diagnosed malignancy in men, claiming over 350,000 lives worldwide annually. Current diagnosis relies on prostate-specific antigen (PSA) testing, but this misses some aggressive tumours, and leads to the overtreatment of non-harmful disease. Hence, there is an urgent unmet clinical need to identify new diagnostic and prognostic biomarkers. As prostate cancer is a heterogeneous and multifocal disease, it is likely that multiple biomarkers will be needed to guide clinical decisions. Fluid-based biomarkers would be ideal, and attention is now turning to minimally invasive liquid biopsies, which enable the analysis of tumour components in patient blood or urine. Effective diagnostics using liquid biopsies will require a multifaceted approach, and a recent high-profile review discussed combining multiple analytes, including changes to the tumour transcriptome, epigenome, proteome, and metabolome. However, the concentration on genomics-based paramaters for analysing liquid biopsies is potentially missing a goldmine. Glycans have shown huge promise as disease biomarkers, and data suggests that integrating biomarkers across multi-omic platforms (including changes to the glycome) can improve the stratification of patients with prostate cancer. A wide range of alterations to glycans have been observed in prostate cancer, including changes to PSA glycosylation, increased sialylation and core fucosylation, increased O-GlcNacylation, the emergence of cryptic and branched N-glyans, and changes to galectins and proteoglycans. In this review, we discuss the huge potential to exploit glycans as diagnostic and prognostic biomarkers for prostate cancer, and argue that the inclusion of glycans in a multi-analyte liquid biopsy test for prostate cancer will help maximise clinical utility.

## 1. Introduction

Prostate cancer is the most common cancer in men, and is a major clinical burden [1]. In the last decade, research into prostate cancer has advanced rapidly, and large advances have been made in both finding new treatments and understanding the underlying biology. However, several areas of urgent unmet clinical need remain, including the identification of: (i) validated biomarkers to complement prostate-specific antigen (PSA) for screening; (ii) prognostic biomarkers with the clinical utility to distinguish indolent and aggressive disease; (iii) molecular stratification methods and predictive biomarkers; and (iv) surrogate end point biomarkers that are valid measures of therapeutic response and survival [2]. As prostate cancer is a heterogeneous and multifocal disease, it is likely that multiple biomarkers will be needed to guide clinical decisions. Fluid-based biomarkers would be ideal, and attention is now turning to minimally invasive liquid biopsies, which enable the analysis of tumour components in patient biological fluids such as blood and urine. Urine is an amenable bodily fluid for prostate cancer biomarker discovery. It is easily obtained in a non-invasive way, and due to the proximity of the prostate to the bladder, urine may carry markers that reflect the development and progression of prostate cancer [3]. Proximal fluids of the prostate such as expressed prostatic secretions (fluid secreted by the prostate after a digital rectal prostate massage) can also be collected in urine and used as a source of biomarkers [4]. Whereas traditional tissue biopsies only provide limited snapshots of the tumour and might fail to reflect heterogeneity, liquid biopsies can provide a comprehensive view of all cancerous lesions (primary and metastases) as well as offering the opportunity to track tumour evolution [5].

A 2018 high-profile review in *Nature Genetics* highlighted that effective diagnostics using liquid biopsies will require the multiparametric analysis of several analytes (including changes to the transcriptome, epigenome, proteome, and the metabolome) within the same blood or urine sample [6]. The detection of changes to the glycome, and more specifically cancer-associated glycan sugar groups, was not included in this review. Glycans have shown huge promise as diagnostic biomarkers for cancer [7,8,9], and recent data suggests that integrating biomarkers across multi-omic platforms (which includes changes to the glycome) can improve the stratification of patients with prostate cancer [10]. 

**Figure 1:**
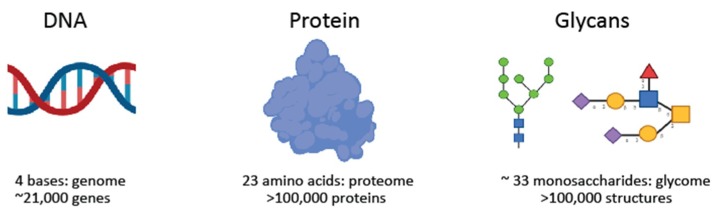
Glycans, the understudied major building blocks of life. The glycoproteome combines three highly adaptable interdependent biological alphabets. Created with BioRender.

**Figure 2:**
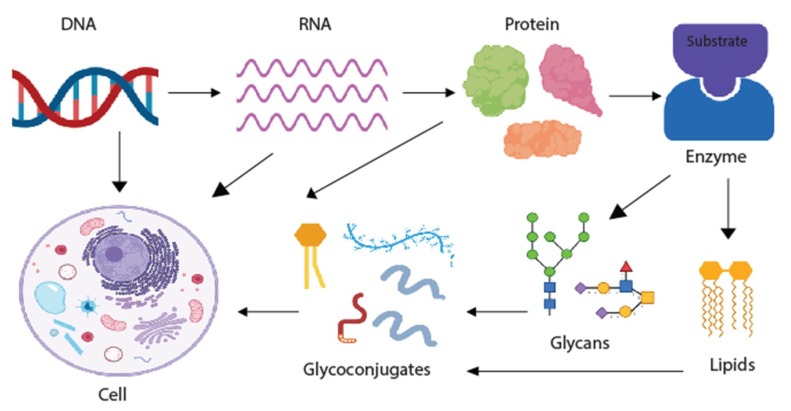
The importance of glycans can be conceptualised as an extended model of the central dogma. Created with BioRender.

Glycans are saccharides that can be attached to proteins, lipids, and other glycans through the enzymatic process known as glycosylation. Glycosylation is the most common posttranslational modification, and is now known to be essential to virtually every biological process in the body [11]. Glycans can either be added sequentially to the hydroxyl oxygen of serine/threonine residues on the target protein (O-linked glycosylation), or as pre-assembled blocks of 14 sugars that are transferred co-translationally via the amide group of an asparagine residue on the target protein (N-linked glycosylation). How much a protein or lipid is glycosylated depends on the number of glycosylation sites present, and the expression of specific glycosylation enzymes within the cell [9]. Glycans are major building blocks of life [12], but have been hugely understudied (Figure 1). This is partly due to their complexity, the difficulties studying them, and that there is no clear link between glycans and DNA. However, this is now changing, and the importance of glycans can be conceptualised as an extended model of the central dogma (Figure 2). The technology to study glycans is rapidly advancing, and there is expected to be an explosion of interest in this area in the next 10 to 15 years, which will be helped by projects such as the human glycome project [13].

Changes to glycans in cancer cells were first described 50 years ago [14], and confirmed by the development of antibodies against tumour-specific antigens [15]. Aberrant glycosylation is a universal feature of cancer cells, and cancer-associated glycans have been detected in virtually every cancer type [16,17]. Cancer cells have numerous changes to glycans compared to normal tissue, including changes to sialylation, fucosylation, the truncation of O-glycans, and N-glycan branching (see [18] for more details). The importance of glycans in cancer is emphasised by the majority of the Food and Drug Administration (FDA)-approved tumour markers being glycan antigens or glycoproteins [19,20,21]. Glycans have key roles in fundamental molecular and cell biology processes in cancer biology, including cell signalling, tumour cell invasion, immune modulation, angiogenesis, interactions with the cell matrix, and metastasis, which are linked to all of the recognised cancer hallmarks [17]. Glycans secreted by cancer cells likely represent the altered glycosylation status of cancer cells, and are a largely untapped resource of cancer biomarkers. There is a huge potential to exploit glycans to improve early diagnosis, as biomarkers for prognosis and stratification, and as markers of specific therapeutic targets [7]. 

The physiological function of the prostate is to act as a secretory gland, which generates and secretes glycoproteins such as PSA into seminal fluid. As such, the prostate is a major secretor of glycans, and there is an unrealised opportunity to detect aberrant glycosylation patterns in serum and urine, and link this to prostate cancer status [22]. As prostate cancer progresses, the epithelial glands become smaller and more rounded, potentially having a drastic effect on the normal secretory pathways where proteins and lipids are glycosylated. Glycans can influence cell survival, proliferation, and metastasis, and likely play a key role in these processes in prostate cancer [22]. A wide range of alterations to glycans have been observed in prostate cancer, including changes to PSA glycosylation, increased sialylation and fucosylation, increased O-GlcNacylation, the emergence of cryptic and branched N-glyans, and changes to galectins and proteoglycans [22,23,24] (see Tables 1–3 for more details). In this review, we discuss the huge potential to exploit glycans as diagnostic and prognostic biomarkers for prostate cancer, and argue that it will be essential to include glycans as part of a multi-analyte liquid biopsy test.

## 2. PSA Glycosylation

Prostate-specific antigen (PSA) is a glycoprotein enzyme that has been used widely as a biomarker for prostate cancer, but has the disadvantage of low specificity and no prognostic value at diagnosis [25]. PSA screening for prostate cancer has led to the overdiagnosis and overtreatment of indolent disease, resulting in unnecessary biopsies and treatments for non-aggressive cancers [26,27]. Refinements on the PSA test, such as the Prostate Health Index (PHI) and 4Kscore, can improve diagnostic accuracy, but have limited prognostic utility, and may miss some high-grade cancers [28]. Thus, there is an urgent clinical need to shift the focus to identifying aggressive disease that needs immediate treatment [29,30,31]. Advances in mass spectrometry have led to an increased interest in glycan structures on cancer-associated proteins, and numerous studies have investigated whether a glycan signature on PSA can be used to improve its clinical utility [21,24]. A recent study also linked a single nucleotide polymorphism that affects PSA glycosylation to prostate cancer risk [32]. PSA has a single N-glycosylation site at asparagine-69 (Asn-69) [24]. Approximately 50 glycoforms of PSA have been described, but only some of these are found in aggressive prostate cancer. In particular, complex biantennary glycoforms with α2,3-sialic acid have been closely linked to aggressive disease in multiple studies [33,34,35] (Table 1). When combined with the PHI, the detection of α2,3-sialic acid PSA glycoforms in a cohort of 79 patients showed 100% sensitivity and 94.7% specificity to differentiate high-risk prostate cancer from low-risk and benign disease [34]. The robust prediction power of α2,3-sialylated PSA to diagnose aggressive prostate cancer has been confirmed by other studies using independent cohorts and different technologies [35]. Together, these studies provide strong evidence in support of the clinical utility of PSA glycoforms to distinguish indolent and aggressive prostate cancer.

## 3. Sialyled Glycans

Sialylation is the process by which sialic acid residues are added to glycans as the terminal monosaccharide. Abnormal sialylation is a universal feature of cancer cells that is linked to poor prognosis and metastasis [9,36,37,38,39,40]. In prostate cancer, sialic acid levels have been investigated as an adjunct to PSA in predicting prostate malignancy [41,42]. Increased levels of α2-3-linked sialic acid have been detected on serum glycoproteins in patients with prostate cancer compared to benign prostatic hyperplasia (BPH), and can be used to predict Gleason score with a higher specificity and sensitivity than PSA [43,44]. Serum sialic acid levels have also been linked to pathological grade, and elevated sialic acid may predict bone metastasis [45]. Together, these studies suggest serum sialic acid as a promising diagnostic biomarker for prostate cancer that should be further investigated for use in predicting disease progression.

An emerging tool for glycoproteomic analysis is the use of azide sugar analogs as mimics of sialic acid. This strategy utilises metabolically labelled glycans with chemical reporters that are ligated to fluorescent probes, and offers a new avenue for probing changes to the glycome by both imaging and glycoproteomic analyses [24,46]. In prostate cancer, sialic acid analogs have been used to identify sialoglycoproteins linked to metastatic potential in cell lines derived from PC3 cells [47]. More recently, sugar azide analogs have been utilised in prostate cancer tissue slices to identify glycoproteins that are elevated or uniquely found in prostate cancer cells [48]. 

As well as changes to serum sialic acid levels and sialoglycoproteins, tumour-associated sialylated glycans also change in prostate cancer. Numerous studies have reported the upregulation of the sialylated blood group antigen Sialyl Lewis X (SLe^X^) in prostate cancer, and linked this with poor prognosis in patients [49,50,51,52] (Table 1). SLe^X^, and its isomer SLe^A^, are the minimal recognition motif for ligands of selectins, which is a family of lectins with roles in leukocyte trafficking and tumour extravasation [53]. SLe^X^ could influence prostate cancer progression through numerous mechanisms, including binding to E-selectin, the evasion of Natural Killer (NK) cell immunity, and the promotion of bone metastasis [52,54,55,56]. The upregulation of SLe^X^ has been detected on PSA, Mucin 1 (MUC1), and prostatic acid phosphatase (PAP) proteins in a panel of 10 malignant tissues (relative to matched normal tissue), opening up a new avenue for the development of prostate cancer-specific glycoprotein biomarkers [57]. The cancer-associated sialyl-Tn glycan (known as sTn) has also been linked to prostate cancer. sTn is a truncated O-glycan containing a sialic acid α-2,6 linked to GalNAc α-O-Serine/Threonine (Ser/Thr) (Table 1). The sTn glycan is upregulated in several cancer types and associated with metastasis and poor prognosis [37]. In prostate cancer, sTn is expressed in high-grade prostate tumours [58,59], and can reduce prostate cancer cell adhesion [60,61]. The sTn antigen is being widely investigated as a circulating biomarker for other cancers [62,63], and it is likely that innovative tools that were developed to detect sTn [63,64,65] will also have clinical utility for prostate cancer precision oncology. 

## 4. Fucosylation

Fucosylation describes the attachment of a fucose residue to a glycan, and consists of terminal fucosylation and core fucosylation. Increased core fucosylation has been detected in the serum of patients with prostate cancer, is associated with disease progression [43,67,68] (Table 1), and may influence prostate cancer cell trafficking through an E-selectin dependent mechanism [56]. The fucosyltransferase FUT8 is the only enzyme responsible for the α-1,6-linked core fucosylation that adds fucose to the inner GlcNAc on N-glycans [76]. FUT8 expression is increased in high-grade and metastatic prostate cancer [73,77]. The increased fucosylation of glycoproteins in aggressive prostate cancer correlates with FUT8 [69], and this has been recently linked to castrate resistance, cell survival, and lower PSA production [78] (Table 1). Taken together, these findings implicate dysregulated fucosylation in prostate cancer progression and the development of castrate resistance. In line with this, several fucosylated glycoproteins are being investigated as potential non-invasive predictive biomarkers. These include decreased fucosylation on PSA [33,79] and integrins [80], and elevated fucosylated haptoglobin [68].

## 5. O-GlcNAcylation

The addition of O-GlcNAc to proteins (known as O-GlcNAcylation) plays a fundamental role in cellular processes, including transcription, epigenetics, cell signalling, proteostasis, and bioenergetics [81,82] (Table 1). In cancer cells, elevated pools of Uridine diphosphate N-acetylglucosamine (UDP-GlcNAc) drive the O-GlcNAcylation of key targets in the cytoplasm, nucleus, and mitochondrion, and can alter key hallmarks of cancer [83,84]. O-GlcNAcylation is catalysed by O-GlcNAc transferase (OGT) (for more details, see [85]). OGT uses UDP-GlcNAc as a substrate, which is produced by the hexosamine biosynthetic pathway (HBP). HBP acts as a sensor for the nutritional state of the cell, and has a key role in the metabolic rewiring observed in cancer [86]. In primary prostate cancer, global O-GlcNAcylation is elevated relative to benign disease [70], and O-GlcNAc levels correlate with a higher Gleason score and reduced patient prognosis [71]. Both OGT and enzymes in the HBP pathway change in prostate cancer. OGT is upregulated in primary prostate cancer, and this is linked to a higher Gleason score, reduced time to biochemical recurrence, and increased c-Myc stability [87]. Similarly, UDP-N-Acetylglucosamine Pyrophosphorylase 1 (UAP1,the last enzyme in the HBP) is also overexpressed in prostate cancer tissue [88]. Although HBP is upregulated in localised prostate cancer where it promotes disease progression, in advanced castrate-resistant disease, the inhibition of HBP enhances tumour growth [72]. Metabolic rewiring during disease progression is believed to promote the downregulation of HBP in castrate-resistant disease, and in particular, loss of the Glucosamine-Phosphate N-Acetyltransferase 1 (GNPNAT1) enzyme may serve as a marker of progression to castrate resistance [72].

## 6. Branched and Cryptic N-Glycans

Changes to N-glycans are common in cancer, and have been linked to metastasis in multiple tumour types [89,90]. Common changes include the branching of complex biantennary glycans to triantennary and tetraantennary structures, and the emergence of cryptic N-glycans [9] (Table 1). In prostate cancer, β-1,6-GlcNAc tri-branched and tetra-branched N-glycans are linked to both metastasis in xenograft models and disease-free survival in patients [73]. Serum N-glycan signatures have shown promise as diagnostic and predictive biomarkers in prostate cancer [91]. Changes to branched N-glycans can help distinguish BPH and prostate cancer [43,92], and increased serum triantennary and tetraantennary N-glycans have clinical utility to predict castrate-resistant prostate cancer [74]. In recent work, a muti-omic study identified tetraantennary and tetrasialylated N-glycans using mass specrometery as part of a biomarker panel to improve the stratification of patients with indolent and aggressive prostate cancer, and predict patient survival [10].

Cryptic N-glycans are the precursors, cores, and internal sequences of N-glycans that are usually masked by other sugar moieties, but occur in cancer due to alterations in glycan synthesis and processing [22,93]. As illustrated in Table 1, they contain the same mannose cores, but differ in the terminal sugar moieties. Cryptic N-glycans expose internal sequences that are normally ‘cryptic’ to the immune system [94]. Several studies have investigated how cryptic N-glycans change among different Gleason grades of prostate cancer and in metastatic disease [73,94,95,96]. Of particular interest, the cryptic N-glycan Man9 has been detected in the serum of men with prostate cancer, and serum Man9 autoantibodies may help differentiate high-grade tumours and predict clinical outcome [75]. The further development and validation of assays to detect anti-Man9 antibodies in patient serum is expected to be useful clinically.

## 7. The F77 Antigen

An antibody against the F77 antigen was initially isolated from mice injected with PC3 cells, and has provoked great interest as a prostate cancer-specific diagnostic and therapeutic tool. F77 was first identified as a glycolipid with α1,2-fucose linkages, and linked to the glycosylation enzymes Fucosyltransferase 1 (FUT1) and Core 2 Branching Enzymes 1-3 (GCNT1, GCNT2, and GCNT3) [97,98,99]. However, F77 has since been detected on O-glycan proteins, including a spliced isoform of CD44 found in the sera of prostate cancer patients [100]. The antibody against F77 can inhibit the growth of PC3 and DU145 tumour xenografts in nude mice, and can be used to differentiate primary and metastatic prostate cancer from non-malignant prostate tissue, [99]. Immunohistochemistry staining tissue sections detected F77 in 112 of 116 primary and 29 of 34 metastatic prostate cancer tissues, while no signal was detected in normal prostate tissue [99].

## 8. Glycolipids

Many of the glycans found attached to proteins are also found on glycolipids (glycolipids are lipids modified by one or more glycans). The aberrant expression of glycolipids in cancer has potential roles in disease progression and anti-tumour immunity (see [101] for more details). In prostate cancer, the F77 antigen (see Section 7 above) is known to contain a glycolipid with α1,2-fucose linkages, and preliminary studies have linked increased ganglioside GD1a to castrate-resistant disease [102,103]. 

## 9. Proteoglycans

Proteoglycans are proteins that are heavily glycosylated. The core protein has one or more glycosaminoglycan (GAG) chains attached (such as chondroitin sulphate, heparin sulphate, or keratin sulphate). Proteoglycans are a major component of the extracellular matrix and interact with growth factors, chemokines, and other extracellular matrix proteins. They have roles in cell signalling, adhesion, cell growth, and apoptosis, and have an established role in cancer progression [104]. Several proteoglycans have been found to be important in prostate cancer, including versican, decorin, biglycan, lumican, and syndecan-1 (summarised in Table 2), and data from a range of studies implicate proteoglycan alterations to prostate cancer cell survival and metastasis [105]. Proteoglycan expression patterns might be useful as predictive and prognostic biomarkers in patients with prostate tumours [22]. Of particular interest, increased levels of versican [106], biglycan [107], and syndecan-1 [108,109] are linked to poor prognosis.

## 10. Galectins

Cancer cells may also display altered expression of proteins that interact with glycans. A key example of this is the galectins, which are a group of glycan-binding proteins with an established role in tumour biology [120]. Several galectins have been implicated in prostate cancer biology (summarised in Table 3). A unique galectin signature has been identified in prostate cancer tissue, with the upregulation of galectin-1 and downregulation of galectins 3, 4, 9, and 12 observed during disease progression [121]. Of particular interest, galectin-3 is linked to tumour progression [122] and has a role in the prostate cancer bone metastasis niche [123]. Immunohistochemistry staining of galectin-3 in tissues from 83 patients had 91.7% sensitivity, 64% specificity, and 73% accuracy in predicting PSA biochemical recurrence [124].

## 11. Upregulation of Glycosylation Enzymes

One of the underlying causes of aberrant glycosylation in cancer is the dysregulated expression of glycosylation enzymes within the cancer cell [16]. Growing evidence links the differential expression of glycosylation enzymes to the progression of prostate cancer [57,78,88,131,132,133,134,135,136] (summarised in Table 4). Androgens are known to drive the development and progression of prostate cancer, and the first line of treatment for men with advanced disease is androgen deprivation therapy (ADT) [137]. Recently, we identified a set of glycosylation enzymes that were regulated by androgens both in seven men undergoing ADT, and in prostate cancer cell lines. Importantly, these enzymes are linked to prostate cancer cell survival, and are upregulated in primary prostate cancer relative to normal and benign tissue [131,132]. The androgen-regulated glycosylation enzymes in prostate cancer include: (i) ST6GalNac1, which synthesises the cancer-associated sTn antigen, (ii) GCNT1, which is linked to the synthesis of sLe^X^, (iii) GalNAc transferase 7 (GALNT7), which is part of a gene signature associated with androgen receptor splice variant-7 (AR-V7), and (iv) the HBP enzyme UAP1, which is highly overexpressed in prostate cancer [57,60,88,132,138]. Several cancer-associated glycans, including sTn, Tn, and sLe^X^ are also androgen regulated in prostate cancer cell lines [132], but this is yet to be studied in primary patient tissue. Preliminary studies have found that GCNT1 can be detected in post-digital rectal examination (post-DRE) urine from 35 prostate cancer patients by immunoblotting, and used to predict the extracapsular extension of prostate cancer (the receiver operating characteristic (ROC) curve analysis for GCNT1 was 0.7614, compared to 0.7455 for PSA) [139]. The *UAP1* gene can also be detected in plasma and urine, and can be used as part of a gene panel to predict prostate biopsy results and prognosis [140,141]. Altered expression of the fucosyltransferases FUT6 and FUT8 are important in advanced prostate cancer. FUT6 is upregulated in distant metastases, and may play a role in metastasis to bone [136]. FUT8 expression increases in aggressive and castrate-resistant prostate cancer, and is linked to poor prognosis in patients (these studies were based on cell lines, and a small number of tissue samples and will need repeating in additional larger cohorts) [77,78]. Taken together, these studies suggest that glycosylation enzymes are a promising but yet unexploited resource of diagnostic and prognostic biomarkers for prostate cancer, and may also provide new insights into candidate glycoprotein alterations.

## 12. Exosomes

Exosomes are small vesicles secreted from most cell types that are present in body fluids. There is increasing evidence that exosomes are involved in carcinogenesis, and they are emerging as a rich source of tumour-specific proteins and biomarkers for cancer detection and progression [146]. Exosomes secreted by the prostate are structurally unique, and can be isolated from seminal fluid, tissue, blood, or urine for further analyses, providing a novel and easily isolatable source of tumour-specific proteins [147]. Glycans are important in exosome function [148]. A recent review discussed the potential to exploitexosomes as powerful tools to diagnose aggressive prostate cancer as early as possible, as well as predict patient prognosis and response to treatment [149]. Exosomes are a rich source of glycans [23,150], but are still largely unexplored in prostate cancer. The profiling of N-linked glycans from prostate cancer exosomes indicates a global decrease in large branched triantennary and tetraantennary glycans that reflects clinical status [151]. A better understanding of the presence of glycoproteins and glycan profiles of exosomes could improve current approaches of diagnosis and prognosis. As discussed by Tkac et al., glycan markers are likely to be enriched in exosomes, which will aid in assay design [23].

## 13. Tissue Imaging of Glycans

Changes to glycans can be profiled in clinically relevant tissues using recently developed innovative approaches. A sialyltransferase-based chemoenzymatic histology assay can detect differences between unsialylated glycans in normal, cancerous, and metastatic prostate tissue sections [152], and bioorthoganol labelling has been used to identify sialoglycoproteins in prostate cancer tissue slices [48]. Advances in imaging mass spectrometry mean that glycans can now be identified directly on patient tissue, allowing for analysis of the spatial distribution of glycans. N-glycans can be profiled on formalin-fixed paraffin embedded (FFPE) tissue samples and tissue microarrays (TMAs) using matrix-assisted laser desorption/ionization imaging mass spectrometry (MALDI-IMS) [24,153,154,155]. In this recently developed approach, glycans are released from their protein carrier and analysed directly on tissue. More than 40 individual glycan structures can be detected, and tumour-specific glycans can be grouped relative to histopathology localisations [155]. A major advantage of MALDI-MS imaging is that the best starting material is FFPE tissue, which is widely available in pathology labs. Ongoing work in this emerging area aims to identify tumour-specific glycan biomarker panels indicative of aggressive prostate cancer, as well as identify a second tier of biomarkers by linking glycans back to their original glycoprotein carriers [23]. It is hoped that information on the various types of glycans within cancer tissue can be used in the future to enable the development of lectin-based assays for use in the pathology lab [155].

## 14. A Multi-Omic Liquid Biopsy Test

The development and progression of prostate cancer from localised, organ-confined disease to biochemically progressive and then to metastasis is prolonged. To capture this evolving landscape, serial tissue biopsies are needed, which is difficult and costly to execute in the clinic. In addition, prostate cancer preferentially metastasises to bone, which is a site that is very difficult to sample. Tumour heterogeneity can also lead to aggressive tumours being missed, or underestimation of the tumour landscape [156]. Liquid biopsies offer a potential solution to overcome the practical and technical challenges of the traditional tissue biopsy [157]. The concept of a ‘liquid biopsy’ relies on the principle that cancer cells are shed directly into blood, urine, and other body fluids. These cancer cells can be captured and used to derive information to improve diagnosis, prognosis, or treatment. Liquid biopsies may also capture a more complete representation of a cancer, which encompasses tumour heterogeneity [158].

Flow cytometery techniques were able to detect circulating prostate cancer cells in men with metastatic disease as early as the 1970s [159]. Today, technologies can use bodily fluids to profile genomic mutations, copy number alterations, and obtain information about the tumour transcriptome, epigenome, proteome, metabalome, and glycome. Among prostate cancer liquid biopsies, circulating tumours cells (CTCs) are the most extensively evaluated biomarkers [160], but other clinically relevant phenotypes have also shown promise. These include detection of the androgen receptor variant V7 (AR-V7) [161], circulating miRNAs [162,163,164], TMPRSS2:ERG, and prostate cancer antigen 3 (PCA3) [165,166,167], circulating free DNA (cfDNA) (reviewed in [158]), detection of gene methylation [168,169], and the analysis of tumour-derived exosomes [170].

Individual biomarkers in liquid biopsy can often not accurately predict disease state due to heterogeneity in phenotype across individuals and over time. To address this challenge, effective diagnostics using liquid biopsies will require multiparameter strategies to combine information from multiple analytes. A recent study by Murphy et al. suggested that integrating biomarkers across multi-omic platforms (including changes to the epigenome, transcriptome, proteome, and glycome) can improve the stratification of patients with prostate cancer [10]. By the analysis of four types of DNA methylation, four coding and nine non-coding RNAs, 27 peptides, and 13 glycans (in a cohort of 158 radical prostatectomy patients), combined with clinical parameters, it was possible to effectively distinguish indolent and aggressive prostate cancer with area under the ROC curve (AUC) = 0.91 (age, PSA level, Gleason score, and DRE gave an AUC of 0.67). This strongly suggests that multivariate models (built from different -omics data) will lead to superior accuracy over individual markers for prostate cancer diagnosis and disease stratification [10], and together with the data discussed in this review, clearly demonstrate that the inclusion of glycans in a multi-analyte liquid biopsy test for prostate cancer should help improve clinical utility. 

## 15. Conclusions and Future Perspectives

Glycans contribute to many aspects of prostate cancer, and likely represent a huge and largely untapped resource of biomarkers with clinical utility. The summary provided by this review suggests that glycan-based biomarkers should be exploited as powerful tools to diagnose prostate cancer as soon as possible, and to accurately determine tumour aggressiveness and patient prognosis. Liquid biopsies hold great potential as non-invasive assays to monitor tumour heterogeneity and evolution, and select personalised therapy. As new markers are identified, their clinical impact must be translated through assay development, followed by clinical reproducibility and sensitivity testing [171]. These efforts raise new biological and technical challenges [158,172], yet constitute critical steps towards precision oncology. The ultimate goal in the field will be to clinically validate several types of molecules, including glycans, within a multi-analyte liquid biopsy test. Furthermore, looking towards the future, artificial intelligence-based tools may be useful to automatically discover and detect these signatures and move the field towards multiparametric analyses [173]. Alongside this, the development of novel glycan-targeting drugs (for examples, see [174,175,176,177,178,179]) will likely lead to new personalised glycan-based therapies.

## Figures and Tables

**Table 1:** Summary of glycan alterations in prostate cancer. PSA: prostate-specific antigen.

Glycan	Structure	Link to Prostate Cancer	References
Example of N-glycan on PSA	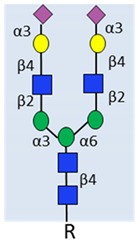 α2-3 sialylated N-glycan	Complex biantennary glycoforms with α2,3-sialic acid have been closely linked to aggressive disease in multiple studies.	[33,34,35].
Sialyl Lewis X (SLe^X^)	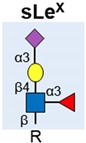	Upregulated and linked to poor prognosis in patients. Detected on PSA, MUC1 and PAP in malignant tissue	[49,50,51,52,57].
Sialyl Tn (sTn)	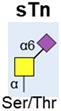	Expressed in high-grade prostate tumours. Can reduce prostate cancer cell adhesion.	[58,59,60,61]
Core Fucosylation	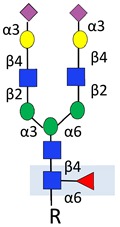	Increased in patient serum. Linked to aggressive disease.	[43,66,67,68]
		Levels correlate with Fucosyltransferase 8 (FUT8).	[69]
O-GlcNAcylation	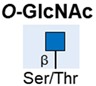	Upregulated and linked to poor prognosis in primary prostate cancer.	[70,71]
		Downregulated in castrate resistant prostate cancer(CRPC).	[72]
Branched N-glycans	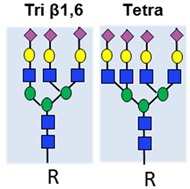	Linked to metastasis and disease-free survival.	[73]
		Predictive biomarker for CRPC.	[74]
Cryptic N-glycans	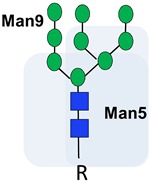	N-linked oligomannose 9 (Man9) is increased in high-grade tumours and linked to clinical outcome.	[75]

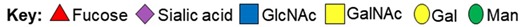

**Table 2:** Proteoglycans with roles in prostate cancer.

Proteoglycan	Link to Prostate Cancer	References
Versican	Modulates binding to the extracellular matrix (ECM) and enhances motility. Associated with poor prognosis.	[106,110,111,112]
Decorin	Suppresses tumour growth by inhibiting both androgen receptor (AR) and epidermal growth factor (EGF). Decreased in prostate cancer.	[106,113,114]
Biglycan	Associated with poor prognosis and PTEN deletion.	[107]
Lumican	Lumican in stroma tissue suppresses cancer progression. Potential marker in prostate cancer staging.	[115]
Perlecan	Linked to disease progression. Upregulates sonic hedgehog signalling.	[116,117]
Syndecan-1	Role in the epithelial-to-mesenchymal transition (EMT). Maintains stability of prostate tumour initiating cells.	[118,119]
	Poor prognosis.	[108,109]

**Table 3:** Galectins with roles in prostate cancer.

Galectin	Link to Prostate Cancer	References
Galectin-1	Upregulated during disease progression. Linked to angiogenesis.	[121]
	Potential therapeutic target in CRPC.	[125,126]
Galectin-3	Promotes prostate tumour growth and invasion. Potential diagnostic marker.	[127]
	High in early stages, lost in advanced disease. May predict biochemical recurrence.	[123,124]
	Role in bone metastasis niche.	[122]
Galectin-4	Linked to metastasis and reduced survival.	[128]
	Part of O-glycosylation-mediated signalling circuit drives metastatic CRPC.	[129]
Galectin-8	Linked to metastasis. Proposed as prognostic biomarker.	[130]

**Table 4:** Summary of glycosylation enzymes with roles in prostate cancer. HBP: hexosamine biosynthetic pathway.

Glycosylation Enzyme	Role in Glycosylation	Link to Prostate Cancer	References
ST6GALNAC1	Transfers α-2,6 sialic acid to O-linked GalNAc	Regulated by androgens. Upregulated in tumour tissue. Linked to the synthesis of sTn. Reduced cell adhesion	[60,61,142]
GCNT1	Forms core-2-branched O-linked glycans	Increased in aggressive disease. Closely related to extraprostatic extension and lymph node metastasis. Increases tumour growth on orthotopic inoculation into the mouse prostate.	[133,134]
		Resistance to NK cell immunity.	[52]
		Regulated by androgens	[132]
		Associated with higher levels of core 2 O sLex in PSA, PAP, and MUC1	[57]
		Linked to F77 antigen.	[97]
		Detected in post-DRE urine. Indicator of extracapsular extension	[139]
GCNT2	Forms core-2-branched O-linked glycans	Linked to invasion. Potential role in integrin signalling.	[143]
GALNT7	Initiation of O-glycosylation	Upregulated in malignant PCa as part of a glycosylation gene signature.	[144]
		Androgen regulated and linked to prostate cancer cell viability.	[132]
		Correlates with AR-V7 in CRPC.	[138]
C1GALT1	Generates the common core 1 O-glycan structure	Part of O-glycosylation mediated signalling circuit that drives CRPC and is linked to poor survival.	[129]
ST6Gal1	Addition of sialic acid to galactose-containing N-glycan	Upregulated. Linked to reduced survival and metastasis.	[135]
		Regulated by androgens.	[132]
EDEM3	Mannose trimming of N-glycans	Upregulated in malignant prostate cancer as part of a glycosylation gene signature.	[144]
		Androgen regulated and linked to prostate cancer cell viability.	[132]
MGAT5	Biosynthesis of β1,6 GlcNAc-branched N-glycans	Link to metastasis in mouse models.	[145]
UAP1	Last enzyme in HBP pathway. Produces UDP-GlcNAc	Highly overexpressed (correlates negatively with Gleason score). Linked to increased UDP-GlcNAc. Protects prostate cancer cells from endoplasmic reticulum (ER) stress. Regulated by androgens.	[88]
GNPNAT1	HBP pathway. Produces UDP-GlcNAc	GNPNAT1 is decreased in CRPC.	[72]
FUT6	Fucosylation	Upregulated in distant metastases. Role in prostate cancer metastasis to bone.	[136]
FUT8	Transfers fucose to core-GlcNAc of the N-glycans	Increased in aggressive prostate cancer and linked to poor prognosis.	[77]
		Increased in CRPC.	[78]
